# Does Parenting Style Affect Adolescent IBD Transition Readiness and Self-Efficacy Scores?

**DOI:** 10.3390/children8050367

**Published:** 2021-05-04

**Authors:** Lynsey R. Zuar, Kimberley Chien, Jennifer Lentine, Victoria Cooley, Linda M. Gerber, Mary J. Ward, Laurie Keefer

**Affiliations:** 1 Division of Pediatric Gastroenterology & Nutrition, Weill Cornell Medicine, New York, NY 10021, USA; kac9091@med.cornell.edu (K.C.); jel2040@med.cornell.edu (J.L.); 2 Clinical & Translational Science Center, Weill Cornell Medicine, New York, NY 10021, USA; vkc4001@med.cornell.edu (V.C.); lig2002@med.cornell.edu (L.M.G.); 3 Development-Behavioral Pediatrics, Weill Cornell Medicine, New York, NY 10021, USA; mjward@med.cornell.edu; 4 Susan and Leonard Feinstein IBD Clinical Center, Icahn School of Medicine at Mount Sinai, New York, NY 10029, USA; laurie.keefer@mssm.edu

**Keywords:** inflammatory bowel disease, adolescent transition, parenting style, transition

## Abstract

Background: Transition to adult-centered care requires adolescents with inflammatory bowel disease (IBD) to acquire a set of independent self-management skills. Transition success can be affected by maturity, cognitive development, and many other factors. Our hypothesis was that parenting style would be associated with increased self-efficacy and therefore transitions readiness. Methods: A prospective cohort survey study of adolescents with IBD and their parents from October 2018 to October 2019 was performed. Participants completed the IBD-Self-Efficacy Scale- Adolescent questionnaire (IBD-SES-A) and the Transition Readiness Assessment Questionnaire (TRAQ). Parents completed the Parent Styles and Dimensions Questionnaire (PSDQ-short form). Demographic and disease information were also collected. Results: Sixty-nine participants were included for full analysis (36 males and 33 females); mean age was 18.2 years, and average age of IBD diagnosis 13 years. Overall, 83% of participants were non-Hispanic Caucasian, and 84% reported parental annual income over USD 100,000. All 69 parents reported an authoritative parenting style. Females have significantly higher TRAQ scores than males (*p* = 0.0004). TRAQ scores differed significantly between age groups, with 20 to 22 years old having higher scores (*p* ≤ 0.0001). TRAQ and IBD-SES-A scores did not differ by parental education or parenting style. Conclusion: Given the inability to delineate different parenting, this study was unable to demonstrate a protective parenting style associated with better transitions readiness and self-efficacy scores in adolescents with IBD. Within the context of authoritative parenting, we did find that females and older adolescents had higher transition readiness scores. Additional research into psychosocial determinants of transition readiness, and the importance of multidisciplinary management with an integrated team including psychologist and social workers, can help improve IBD transition outcomes.

## 1. Introduction

Adolescent transition readiness includes providing necessary education on their disease and helping them acquire the self-management skills necessary to function independently in order to successfully transition to the adult health-care system. IBD incidence is rising [1], and 20–30% of patients with IBD are diagnosed before age 20 [2]. Maturity and cognition of individuals differ, and can be affected by chronic disease, therefore transition initiation time can vary. The North American Society for Pediatric Gastroenterology, Hepatology and Nutrition (NASPGHAN) states that successful transition requires collaboration with the patient, family, and healthcare team. There is an acknowledgment that psychosocial dynamics do affect transition readiness in the IBD population. They stress teaching self-management, knowledge of disease, medications and tests, independence, assertiveness, and lifestyle management [3,4]. Historically the focus of disease management has been the pathology of the disease itself rather than the external influences that affect to overall health of the patient. An important aspect to focus on within this population are factors that affect the psychological health of the adolescent patient with IBD, and this ultimately will affect health outcomes and successful transition to adult medicine. It is well known that a body that is undergoing psychological stress can lead to a proinflammatory state and can reduce one’s ability to cope [5]. An integrated IBD transition model includes a multidisciplinary approach between gastroenterologists, surgeons, nurses, dietitians, pathologists, radiologists, and very importantly social workers and psychologists [6]. Psychosocial factors outside the primary disease should be addressed regularly as they will affect the adolescent’s ability to transition.

**Figure 1:**
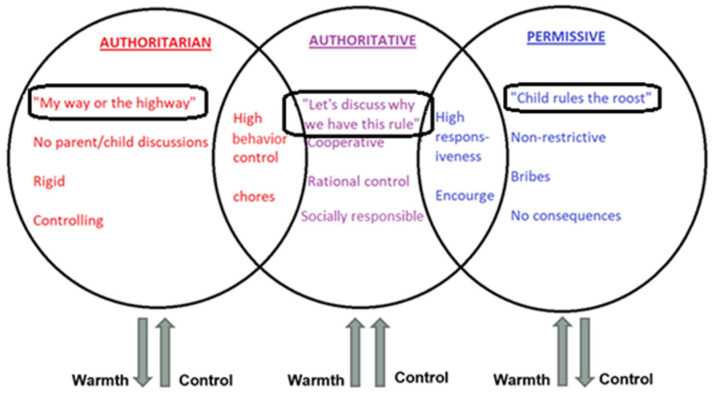
Parenting styles (based on Baumrind parenting styles [7]).

One such important contributor to psychosocial health is parenting style. In 1966, Diana Baumrind Ph.D. categorized parenting style into: authoritative (high amount of control, and warmth with a balance of autonomy), authoritarian (high amount of control, but low warmth), and permissive (high amount of warmth, and low control) [Figure 1] [7]. Studies have shown that authoritative parenting style decreases risky behaviors [8] and helps with management of other chronic diseases such as type 1 diabetes mellitus [9,10,11], and obesity [12,13,14]. The aim of the study was to determine the association between parenting style, self-efficacy scores, and transition readiness scores among adolescent participants with IBD. A secondary aim was to identify demographic factors that are associated with transition readiness in adolescent participants with IBD and use these to identify those who are easier or harder to transition into adult medicine. The literature is limited on how parenting styles affect transition readiness in patients, particularly adolescents with IBD. The original purpose of this study was to see if certain parenting styles were associated with increased self-efficacy and transition readiness scores within the population of adolescents’ with IBD. Could these measures be used as a way to screen for higher or lower risk patients when it came to transition success? Our hypothesis was that an authoritative parenting style would be positively associated with increased transition readiness and self-efficacy scores.

## 2. Materials and Methods

### 2.1. Participants

Adolescent participants with IBD aged 16 to 22 years, along with a designated parent, were enrolled between October 2018 and October 2019 at an urban academic tertiary IBD medical center in Manhattan as part of a prospective cohort survey study. Due to the exploratory and hypothesis generating nature of this study, a formal power calculation was not executed. Our numbers were limited to the patient population of AIBD patients within our institution. For participants under 18 years of age, informed consent and assent were obtained from parents and patients, respectively. For those 18 years of age or older, consent was obtained directly from the participants. Each participant was asked to complete a validated surveys of transition readiness and IBD self-efficacy: The Transition Readiness Assessment Questionnaire (TRAQ) [15], and the IBD Self-Efficacy Scale Adolescent (IBD-SES-A) [16]. Each participant also had to designate a parent (or parental figure/guardian) to complete a parenting measure [17]. IBD diagnosis and demographic information (Table 1) were collected at the time of consent. Exclusion criteria included non-English speaking, cognitive impairment deemed by provider, other serious or psychological illness, and pregnancy and/or emancipated minors. The Weill Cornell Medicine Institutional Review Board approved the study (protocol code 1801018912, date of approval 10 December 2019).

The participants were further subdivided into different groups for analysis. TRAQ scores and IBD-SES-A scores were compared and analyzed between female and male adolescent participants, participants aged 16–19 versus age 20–22, those whose parents had a Bachelor’s degree or below and a Master’s degree or above, and between parents who had a higher or lower authoritative score using the mean authoritative score of 4.3 as the cutoff.

### 2.2. Measures

*The Parenting Styles and Dimensions Questionnaire Short Form* is a 32-item parent reporting questionnaire [17]. The original questionnaire is based on Baumrind’s theory of 3 parenting styles: authoritarian, authoritative, and permissive parenting. Each of the items is rated on a Likert-scale (1 = Never to 5 = Always). A higher averaged score indicates that that parenting behavior type is more frequent. Each question is assigned to either authoritarian, authoritative, or permissive parenting. All questions for the 3 different categories are averaged to get an overall score for each parenting type ranging from 0 to 5. The parenting type with the overall highest averaged score is the participants designated style.

*The Transition Readiness Assessment Questionnaire* is a 20 item five-point Likert-scale questionnaire (1= No, I do not know how to 5 = Yes, I always do this when I need to; range score 20–100) [15]. It is a validated questionnaire used to assess adolescents’ skills needed for transition to adult medical care. There are five domains determining different aspects of self-care: appointment keeping, tracking health issues, managing medications, talking with providers, and managing daily activities. The score is summed with a higher score indicating a higher ability to independently execute medical management. This is a measurement tool for general adolescent transition and is not specific to pediatric gastroenterology. A 2014 study done by Wood et al. measured the reliability and validity of the TRAQ, and showed the overall scale has high reliability overall.

*The IBD Self-Efficacy Scale Adolescent* is a 13 item Likert-scale IBD specific questionnaire (1 = completely disagree to 5 = completely agree) which determines measurements of confidence in self-management tasks related to IBD medical care, and emotional responses in relation to living with IBD [16]. The score is summed, and a higher score indicates higher self-efficacy. Carlsen et al. showed self-efficacy was a better predictor of transition readiness in the adolescent age group [18]. The IBD-SES-A questionnaire helps to measure a set of beliefs about one’s ability to perform certain tasks and scenarios related to their IBD, regardless of the participants’ age.

Participants were also asked to complete a demographics and general health status information worksheet summarized in Table 1. IBD history was extracted from the medical record.

### 2.3. Statistical Analysis

Descriptive statistics using *N* (%) and mean, standard deviation (SD), median, and range were utilized to generate summary information of the demographic and clinical characteristics of the sample. Wilcoxon rank sum tests were used to compare median TRAQ and IBD-SES total sum scores across gender, age group, and parenting style groups. Statistical significance was evaluated at the 0.05 alpha level. All analyses were performed in R (3.5.1) for Windows 10.

## 3. Results

### 3.1. Study Population

One hundred and four participants were approached, and 82 participants were ultimately enrolled, but 13 participants were removed due to incomplete data. Imputation was determined to not be possible since subjects that were removed had all or one of their PSDQ, TRAQ, and/or IBD-SES-A forms missing. A total of 69 participants remained for full analysis once data collection was complete (36 males and 33 females). Of the 69, 10% of participants were diagnosed with ulcerative colitis (UC), and 90% were diagnosed with Crohn’s disease (CD). Average age of IBD diagnosis was 13 years. There were no significant demographic differences between those who enrolled and those who declined, or between those who completed all the surveys and those removed due to incomplete data. Participants had a median age of 18.2 years (min-max: 16–22 years) and were primarily non-Hispanic Caucasian (82.6%). Most participants had a parental annual income over 100,000 dollars a year (84.1%).

### 3.2. Data

**Figure 2:**
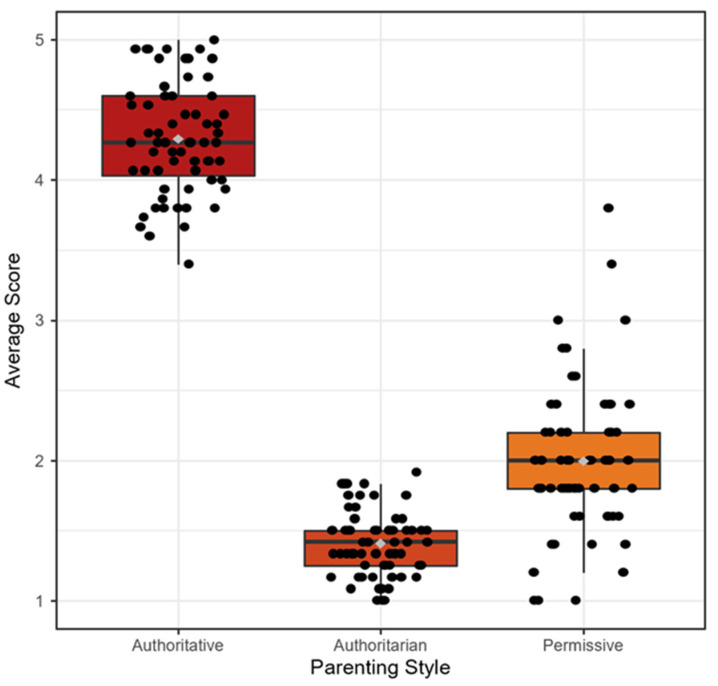
Parenting style score distribution.

*Parenting style*. All 69 parents who completed the PSDQ questionnaire scored highest for the authoritative parenting style. The authoritative PSDQ domain mean distribution showed a mean 4.3 +/− 0.4, median 4.3, and range 3.4–5. The authoritarian PSDQ domain mean distribution showed a mean 1.4 +/− 0.2, median 1.4, and range 1–1.9. The permissive PSDQ mean distribution showed a mean 2 +/− 0.5, median 2, and range 1–3.8 (Figure 2). After initial results showed all participants in the same parenting style, a secondary analysis was conducted to determine if parent’s second highest parenting style type (permissive or authoritarian) was associated with trends in IBD-SES-A and TRAQ scores; there was no statistical significance between the two groups of second highest parenting style.

*Transition Readiness*. The TRAQ total sum score mean was 74.5 (Table 2 (A)). TRAQ scores were higher in female participants when compared to male participants (Table 2 (B), *p* = 0.0004). TRAQ scores between different age groups revealed that the age group of 20–22 years old had a significantly higher score (Table 2 (C), *p* < 0.0001). The difference in mean TRAQ scores for participants whose parents had a less advanced degree versus a more advanced degree was insignificant (Table 2 (D)). TRAQ scores compared between parents who scored higher or lower within the authoritative scores were also not significant using the mean of 4.3 as the cutoff to separate the two groups (Table 2 (E)).

*Self-efficacy*. The IBD-SES-A total sum score mean was 53.2 (Table 3 (A)). IBD-SES-A scores were higher in female participants when compared to male participants but were not statistically significant (Table 3 (B)). IBD-SES-A scores between different age groups (20–22 years old vs. 16–19-year-old) did not show significance (Table 3 (C)). The difference in mean IBD-SES-A scores for participants whose parents had a less advanced degree versus a more advanced degree was insignificant (Table 3 (D)). IBD-SES-A scores compared between parents who scored higher or lower within the authoritative scores were also not significant, using the mean of 4.3 as the cutoff to separate the two groups (Table 3 (E)).

## 4. Discussion

Ultimately, all the parents who participated in our study did end up self-reporting an authoritative parenting style. This may have been due to sampling, social desirability, or recall bias. It also may highlight an overall generational trend towards an authoritative parenting style as seen in a 2007 Australian study [19]. A significant limitation of this study included the small sample size, decreasing the power of the data, as well as a lack of diversity in our study population; most of the participants were affluent, non-Hispanic white, with 84% of the families having a yearly income of USD 100,000 or more.

While comparing other demographic measures within our patient cohort to both TRAQ and IBD-SES-A scores, we were able to again highlight higher TRAQ scores in females, which was previously identified by McManus et al. for the transition of patients with special needs (including chronic medical conditions) [20]. Although this was only found within authoritative parenting style, the female gender as a protective factor for transition readiness in the adolescent IBD population warrants further investigation. If gender is truly a good predictor of adolescent IBD transition outcomes, this can be used to promote more self-reliance during visits with male patients’ earlier in adolescence. Our study did also show that TRAQ scores differed significantly between age groups, with 20 to 22 years old having higher scores than the younger group. However, as seen with previous studies, the IBD-SES-A scores were not affected by participants’ age, and thusly might be a more reliable screen to measure future success of transition in our younger adolescent patients with IBD [21]. It would be worth investigating how gender, and age affect transition measures (TRAQ and IBD-SES-A scores) in a larger, more diverse IBD population.

Pediatric IBD management, as with any pediatric chronic disease, requires a multidisciplinary team approach. The medical community can no longer only focus on the disease process itself and hope to have success in overall IBD treatment and transition. Psychosocial factors, such as a parent and child relationships, can affect a patient’s ability to manage their IBD. It is important to have a strong psychology and social work presence as part of an integrated team approach to help guide adolescent IBD patients though management of disease and transition into adult medicine. This area of focus has great potential to improve disease outcome and transition success since pediatric physicians are already aware that transition to adult medicine occurs during a period when adolescents’ with IBD are at increased risk for bad health outcomes [18]. There are many complex social issues such as the parent-child relationship that affect an adolescent’s ability to be resilient and self-sufficient, and ultimately their ability to successfully transition to adult medicine specialties [22,23].

Parenting styles have been studied in other pediatric chronic diseases, but more studies are needed to see how this can affect transition readiness of adolescents with IBD. Since parenting style is a foundation for a persons’ beliefs, can shape self-efficacy skills, and helps define future values, it makes sense that previous research shows authoritative parenting style having a positive outcome in other pediatric chronic diseases [9,10,11,12,13,14]. Authoritative parents’ guide their children with a good balance of support, love, and boundaries, and this parenting style leads to less risk taking related to substance abuse disorders [8]. It is also associated with better glucose control in type 1 diabetes mellitus [9], and decrease rates of obesity [12,13,14]. At present, the literature is limited on how parenting style affects transition readiness in patients with IBD, and this was a novel study to compare parenting style scores with an IBD specific transition measure of self-efficacy (IBD-SES-A), along with a frequently used transition measure (the TRAQ).

## 5. Conclusions

We were unable to support our hypothesis and identify whether a protective parenting style is associated with better transitions readiness and self-efficacy scores in adolescents with IBD. Within the context of authoritative parenting, we did find that females and older adolescents had higher transition readiness scores. This research study also highlighted that female adolescent participants with IBD within the authoritative parenting style had higher transition readiness scores. Additional research into determinants of transition readiness in IBD is needed to optimize care for this vulnerable population. Our study highlighted that more research is needed to fully understand how various aspects of psychosocial health affects transition readiness in the adolescent IBD population. Another important takeaway from this study is highlighting the importance of a multidisciplinary team, including psychologist and social workers, who can help guide the psychological health required for successful transition readiness in adolescents with IBD.

## Figures and Tables

**Table 1:** Demographic information.

**Age Ranges: *N* (%)**	
16–19:	47 (74.6)
20–22:	16 (25.4)
**Age at diagnosis: Mean (Range)**	12.95 (11–17)
**Gender: *N* (%)**	
Male:	34 (53.97)
Female:	29 (46.03)
**IBD type: *N* (%)**	
CD:	56 (88.89)
UC:	7 (11.11)
**Race/Ethnicity: *N* (%)**	
Non-Hispanic White:	52 (82.54)
Hispanic:	3 (4.76)
African American:	3 (4.76)
Asian/Pacific Islander:	1 (1.59)
Native American/Aleut:	0 (0)
Other:	4 (6.35)
**Highest Level of Schooling Completed: *N* (%)**	
Currently in HS:	24 (38.10)
HS graduation or equivalent:	3 (4.76)
Currently in college:	32 (50.79)
Graduated college:	3 (4.76)
Currently in post-graduate school:	1 (1.59)
**Parental Highest Level of Education: *N* (%)**	
HS graduate or equivalent:	3 (4.76)
Some college credit:	6 (9.52)
Bachelor’s degree:	22 (34.92)
Master’s degree:	23 (36.51)
Advanced Graduate or Ph.D.:	9 (14.29)
**Parent’s yearly household income: *N* (%)**	
<USD 25,000:	0 (0)
USD 25,000–49,999	1 (1.59)
USD 50,000–74,999	2 (3.17)
USD 75,000–99,999	7 (11.11)
>USD 100,000	53 (84.13)

CD = Crohn’s disease; UC = ulcerative colitis.

**Table 2:** TRAQ score comparisons.

	***N***	**Mean**	**Median**	**Min**	**Max**
**A. TRAQ total**	69	74.51	75	36	100
**B. Gender**	***N***	**Mean**	**Median**	**Min**	**Max**
Male	36	68.10	66	36	97
Female	33	81.55	84	53	100
**C. Age Groups**	***N***	**Mean**	**Median**	**Min**	**Max**
16–19 yo	52	70.02	69	36	100
20–22 yo	17	88.24	86	74	100
**D. Parent Education**	***N***	**Mean**	**Median**	**Min**	**Max**
Bachelor’s	35	73.43	72	38	100
or below					
Master’s	34	75.62	78.5	36	100
or above					
**E. Higher and Lower** **Authoritative Score**	***N***	**Mean**	**Median**	**Min**	**Max**
Higher score	32	75.78	76	36	100
Lower score	37	73.41	75	38	97

**A.** TRAQ total score sum. **B.** TRAQ gender groups comparisons: *p* = 0.0004, there is sufficient evidence to conclude total TRAQ score differs between the 2 genders. **C.** TRAQ score and age groups comparisons: *p* < 0.0001, there is sufficient evidence to conclude total TRAQ score differs between the 2 groups. **D.** TRAQ education groups comparisons (Bachelor’s and below vs. Master’s and above): *p* = 0.4248, insufficient evidence to conclude total TRAQ score differs between the 2 education groups. **E.** TRAQ parenting style comparisons (using mean as cut-off): *p* = 0.5004, insufficient evidence to conclude that the median total TRAQ score differs between high and low scoring authoritative groups.

**Table 3:** IBD-SES-A score comparisons.

	**N**	**Mean**	**Median**	**Min**	**Max**
**A. IBD-SES-A total**	69	53.23	53	37	65
**B. Gender**	**N**	**Mean**	**Median**	**Min**	**Max**
Male	36	51.97	52	37	65
Female	33	54.61	54	46	64
**C. Age Groups**	**N**	**Mean**	**Median**	**Min**	**Max**
16–19 yo	52	70.02	69	36	100
20–22 yo	17	88.24	86	74	100
**D. Education**	**N**	**Mean**	**Median**	**Min**	**Max**
Bachelor’s or below	35	52.71	52	42	64
Master’s or above	34	52.76	55	37	65
**E. Higher and Lower Authoritative Score**	**N**	**Mean**	**Median**	**Min**	**Max**
Higher score	32	52.5	53	37	62
Lower Score	37	53.5	53	41	65

**A.** IBD-SES-A total sum score. **B.** IBD-SES-A gender groups comparisons: *p* = 0.096, there is insufficient evidence to conclude total TRAQ score differs between the 2 genders. **C.** IBD-SES-A score and age groups comparisons: *p* = 0.274, there is insufficient evidence to conclude total TRAQ score differs between the 2 groups. **D.** IBD-SES-A education groups comparisons (Bachelor’s and below vs. Master’s and above): *p* = 0.3777, insufficient evidence to conclude total IBD-SES-A score differs between the 2 education groups. **E.** IBD-SES-A parenting style comparisons (using mean as cut-off): *p* = 0.4448, insufficient evidence to conclude that the median total IBD-SES-A score differs between high and low scoring authoritative groups.

## Data Availability

The data that support the findings of this study are available on request from the corresponding author, [L.R.Z.]. The data are not publicly available due to their containing information that could compromise the privacy of research participants.
